# Cerebrospinal fluid leakage after retrosigmoid craniotomy and craniectomy for cerebellopontine angle tumors: A comparative retrospective study

**DOI:** 10.1097/MD.0000000000042838

**Published:** 2025-06-13

**Authors:** Min Tang, Seidu A. Richard, Zhigang Lan

**Affiliations:** a Department of Neurosurgery, West China Hospital, Sichuan University, Chengdu, Sichuan, P. R. China; b Institute of Neuroscience, Third Affiliated Hospital, Zhengzhou University, Zhengzhou, China; c Department of Biochemistry and Forensic Sciences, School of Chemical and Biochemical Sciences, C. K. Tedam University of Technology and Applied Sciences (CKT-UTAS), Navrongo, Ghana; d Department of Neurosurgery, West China Xiamen Hospital, Sichuan University, Xiamen, Sichuan, P. R. China.

**Keywords:** CPA, craniectomy, craniotomy, CSF, leakage, watertight

## Abstract

The incidence of CSF leakage after surgery is very critical in the management of patients with cerebellopontine angle (CPA) tumors. The aim of our current study is to compare the outcome of retrosigmoid craniotomy and craniectomy in terms of the rate of postoperative CSF leakages and burdens such as in-hospital stay and cost on patients. In this comparative retrospective study, data was retrieved to identify the patients who underwent retrosigmoid craniotomy and craniectomy from January 2019 to December 2022 at Department of Neurosurgery, West China Hospital. Data such as age, sex, preoperative hydrocephalus, tumor type and sizes, length of in-hospital stay, cost, and intracranial infection were documented and compared. The primary outcome was development of postoperative CSF leakage. A total of 345 patients were retrieved, out of which 83 (24%) patients underwent craniotomy while 262 (76%) underwent craniectomy. The incidence of postoperative CSF leakage was significantly lower in the craniotomy group compared to the craniectomy group (*P* = .0059). Similarly, craniotomy was superior in terms of in-hospital stay (*P* < .0001) and average cost (*P* < .0001) compared to craniectomy. Craniotomy reduces incidence CSF leakage in cases where the dura could not be sutured in a watertight fashion compared to craniectomy. Also, craniectomy puts more burden on patients compared to craniotomy.

## 1. Introduction

Cerebellopontine angle (CPA) tumors occur in the region between the cerebellum and the pons.^[1,2]^ These tumors present with various symptoms such as hearing loss, tinnitus, vertigo, and facial weakness.^[1,3,4]^ The retrosigmoid approach is a surgical technique used to access lesions at posterior fossa of the brain.^[5]^ It is commonly used for the removal of tumors located in the CPA.^[5,6]^ However, the surgery may result in dura defects that often leads to cerebrospinal fluid (CSF) leakage.^[7]^ The dura is a tough membrane that covers the brain and spinal cord.^[8]^ It acts as a protective barrier and prevents the leakage of CSF from the brain and spinal cord.^[8]^ However, in some cases, due to dehydration, surgical tear and adhesion especially in older patients, the dura cannot be primarily sutured in a watertight fashion after the surgery.^[9,10]^

Notably, CSF leakage after retrosigmoid approach is still a challenge for many neurosurgeons due to complications and prolong the recovery time of the patient.^[7,11]^ Also, despite the use of varying allografts such as fascial, fat, and or muscle autografts, postoperative lumbar drainage, and pressure or vacuum dressings CSF leakages are still not completely resolved postoperatively.^[9,12–15]^ Craniotomy is a surgical procedure that involves making an incision in the scalp and drilling a hole in the skull to get access to the brain.^[9,16]^ The bone flap is then replaced at the end of the surgery.^[17]^ Craniectomy, on the other hand, involves removing a portion of the skull and not replacing it back after the surgery to allow for swelling of the brain.^[9,16,18]^ Both procedures have their advantages and disadvantages and are frequently used in retrosigmoid approach.^[7]^

Previous studies observed difference in terms of CSF leak between craniotomy and craniectomy when the primary dura closures were not completely achievable in posterior fossa.^[9]^ Thus, the replacement of bone flap may play a vital role in the prevention of CSF leakage, especially in cases where original dura cannot be tightly sutured and even with the use of various dura grafts to seal the defects. The aim of our current study is to compare the outcome of retrosigmoid craniotomy and craniectomy in terms of the rate of postoperative CSF leakages during admission up to 3 months after surgery and burdens such as in-hospital stay and cost on patients.

## 2. Methods and materials

In this comparative retrospective study, data was retrieved to identify the patients who underwent retrosigmoid craniotomy and craniectomy from January 2019 to December 2022 at Department of Neurosurgery, West China Hospital, Sichuan University. The institutional review boards of West China Hospital approved this study. The patients and their relatives fully consented to the use of their information after dually informing them about our intention to involve them in a study. All the patients signed written informed consents for publication. Individuals evaluating the chart review from medical records were all trained by the corresponding author to ensure consistency of review and coding.

Data such as age, sex, preoperative hydrocephalus, tumor type and sizes, length of in-hospital stay, cost, and intracranial infection were retrieved from electronic medical records and compared. Also, co-morbidities such as diabetes, hypertension, BMI > 30, malignancy were extracted from preoperative records while radiotherapy and chemotherapy treatments were retrieved from postoperative records. Radiographic reports such as magnetic resonance imaging and computed tomography (CT) reports were used to establish preoperative hydrocephalus. Also, surgical notes were used confirm each surgical approach with particular emphasis on development of CSF leakages (rhinorrhea, otorrhea, pseudomeningocele, and incisional leak) on admission up to the first surgical follow-up. Patients whose dura were sutured in a water tight fashion and without leakage were excluded from the final analysis. The maximum diameter of the craniotomy/craniectomy was measured postoperatively using a computer tool on the CT skull base reconstruction scan.

### 2.1. Surgical technique

Preoperative hydrocephalus was managed with EVD placement 24 to 48 hours before surgery, and removed postoperatively after CSF pressure normalization. In both the craniotomy and craniectomy patients, straight incisions behind their ears were used to open the scalps. In the Craniotomy group, holes were drilled at the junctions of the transverse sinus and sigmoid sinus with a high speedy electric drill. Notably, any additional holes were drilled in the occipital bone if necessary. The bone flaps were fully separated from the venous sinus and dura adhesions after using a high-speed electrical cutter to cut the skull. Also, after resecting the tumors, bone flaps were replaced and fixed by titanium caps or connecting pieces with titanium nails. The straight incisions behind their ears were suture together afterwards.

In the craniectomy group, several holes were drilled near the transverse sinus, sigmoid sinus and occipital bone with a high speedy electric drill. Also, any additional holes were drilled in the occipital bone if necessary. The bone flaps were fully separated from the venous sinus and dura adhesions after using a high-speed electrical cutter to cut the skull. Notably, the bone windows were enlarged with rongeur by biting off the edges of the bone around the sinus. Contrarily, the skull defect was not repaired after tumor resection. Also, the straight incisions behind their ears were suture together afterwards.

In both approaches, the sizes and extent of the bone windows were estimated so as to fully expose the transverse and sigmoid sinus junctions. Also, CSF release, tumor resection, intraoperative facial nerve monitoring, posterior cranial nerve and manipulation techniques such as vascular protection were carried out according to our institution protocol and guidelines. Furthermore, in both approaches, the utmost goal was to achieve total watertight closure of their dura. However, in cases where the dura mater could not be primarily sutured to attain total watertightness, the defects were covered with artificial dura mater or allografts such as fascial, fat, and or muscle autografts, and reinforced with bioprotein glue. For dural defects > 1 cm, fascia lata autografts or synthetic dura (DuraGen®) were used for watertight closure. Dural closure was performed using interrupted 5-0 Prolene sutures at 3 to 4 mm intervals, reinforced with fibrin glue (Tisseel®).

### 2.2. Postoperative complication and CSF leakage

Our primary outcome measure was development of a CSF leakage during the initial hospital stay and 3 months after surgery. Notably, 3 months after surgery is also the first surgical follow-up. In all cases of CSF leakages records were obtained from MRI with gadolinium and high-resolution CT cisternography which are utilized by our institution to detect the location of CSF leakages. Final tumor diagnosis was retrieved from pathology reports. The duration of in-hospital stays and the total cost of treatment of all the patients were retrieved from the patients’ records.

### 2.3. Statistical analysis

Data was entered and preserved for analysis in the SPSS software version 25.0 (IBM Corp., Armonk, New York). The results of Fisher exact test are reported for 2-by-2 tables. Also, the Mann–Whitney test was utilized to calculate the significance of ordinal variables, and the unpaired *t*-test or analysis of variance was utilized for continuous variables. All statistical tests were 2-tailed. *P* < .05 was considered statistically significant.

## 3. Results

### 3.1. Demographic parameters

We retrieved a total of 524 patients who underwent retrosigmoid craniotomy and craniectomy. However, only 345 patients met our inclusion criteria while 179 met our exclusion criteria. Patient characteristics can be found in Table 1. Also, out of the 345 patients who met our inclusion criteria, 83 (24%) patients underwent craniotomy while 262 (76%) underwent craniectomy. Furthermore, the mean age of the patients who underwent craniotomy was 54.5 ± 0.53 while the mean age of the patients who underwent craniectomy was 53.8 ± 0.46. However, there was no significant difference in terms of age between craniotomy and craniectomy groups (*P* = .53). Also, in terms of gender, 45 (13%) patients were males while 38 (11%) patients were females in craniotomy group. On the other hand, in the craniectomy group, 134 (39%) patients were males while 128 (37%) patients were females. However, there was no significant difference in terms of gender between craniotomy and craniectomy groups (*P* = .62). The mean craniotomy diameter was 3.8 ± 0.5 cm, and craniectomy diameter was 4.2 ± 0.6 cm. Multivariate analysis revealed no significant association between bone window size and CSF leakage (*P* = .21) as shown in Table 1.

**Table 1:** Patient demographics.

Parameter	Craniotomy group(N = 83)	Craniectomy group(N = 262)	*P*-value
Age	54. 5 ± 0.53	53.8 ± 0.46	.53
Gender			
Male	45	134	.62
Female	38	128	
Preoperative hydrocephalus	2	5	.67
Tumor size	3.8 ± 0.23	4.1 ± 0.3	.65
Bone window size	3.8 ± 0.5	4.2 ± 0.6	.21
Tumor type			
Acoustic neuroma	36	115	.08
Meningioma	20	78	
Metastasis	10	29	
Glioma	6	20	
Hemangioblastoma	5	13	
Epidermoid cyst	3	1	
Cavernous malformation	2	1	
Choroid plexus papilloma	1	1	

### 3.2. Preoperative hydrocephalus and Surgical Outcome

Notably, 2 patents had preoperative hydrocephalus in craniotomy while 5 patients had preoperative hydrocephalus craniectomy groups. However, there was no significant difference in terms of preoperative hydrocephalus between craniotomy and craniectomy groups (*P* = .67). Also, the mean tumor size in craniotomy group was 3.8 ± 0.23 while the mean tumor size in craniectomy group was 4.1 ± 0.3. However, there was no significant difference in terms of tumor size between craniotomy and craniectomy groups (*P* = .65). The tumor types resected included acoustic neuroma, meningioma, metastasis, glioma, hemangioblastoma, epidermoid cyst, cavernous malformation, and choroid plexus papilloma. The tumor distribution between craniotomy and craniectomy groups is as shown in Table 1. Notably, there was no significant difference in terms of tumor type between craniotomy and craniectomy groups (*P* = .08).

### 3.3. Postoperative CSF leakage, infection, hospital stay, and cost

Interestingly, only 3 patients developed CSF leakages in the craniotomy group while 38 patients developed CSF leakages in craniectomy group. Thus, we observed a significant difference in terms of CSF leakage between craniotomy and craniectomy groups (*P* = .0059). All CSF leakages occurred during the in-hospital stay and no CSF leakages occurred 3 months after surgery or the first surgical follow-up. Also, only 1 patient developed intracranial infection in the craniotomy group while 15 patients developed intracranial infection in craniectomy group. However, there was no significant difference in terms of intracranial infection between craniotomy and craniectomy groups (*P* = .1315) as shown in Table 2. Patients who developed intracranial infections were treated with intravenous antibiotic base on culture and sensitivity results from the laboratory.

**Table 2:** Patient outcome.

Parameter	Craniotomy group (N = 83)	Craniectomy group (N = 262)	*P*-value
CSF leak	3	38	.0059
Intracranial infection	1	15	.1315
Length of stay (d)	9.5 ± 1.6	14 ± 2.1	<.0001
Average cost (RMB)	58,302 ± 34.5	70,399 ± 55.2	<.0001

CSF = cerebrospinal fluid.

Among 7 patients with preoperative hydrocephalus, 1 developed CSF leaks (14.3%), comparable to the non-hydrocephalus group (12.1%, *P* = .71). In terms of co-morbidities, diabetes (*P* = .013) and obesity (BMI > 30, *P* = .038) independent predictors of CSF leakage while malignancy (*P* = .4724) and hypertension (*P* = .1273) were not predictors of CSF leakage as shown in Table 3. In of adjunct therapies, 47 patients (1.4%) underwent postoperative radiotherapy and 2 developed CSF leakage (*P* = .2247), while 37 patients underwent chemotherapy and 3 developed CSF leakage (*P* = .4724) as shown in Table 3.

**Table 3:** Co-morbidities and adjunct therapies with outcome.

Confounding factors	CSF leak (n = 41)	No CSF leak (n = 304)	*P*-value
Diabetes	12 (29.3%)	45 (14.8%)	.013
BMI > 30	9 (22.0%)	32 (10.5%)	.038
Malignancy	2 (4.9%)	10 (3.3%)	.4724
Hypertension	4 (9.8%)	19 (6.3%)	.1273
Radiotherapy	2 (4.9%)	45 (14.8%)	.2247
Chemotherapy	3 (7.3%)	34 (11.2%)	.4724

BMI = body mass index, CSF = cerebrospinal fluid.

Nature of CSF leakages (Table 4) included pseudomeningocele (30/41, 73.2%), wound leakage (8/41, 19.5%), and rhinorrhea (3/41, 7.3%). No otorrhea was observed. CSF leakages were managed (Table 4) with lumbar drainage (35/41, 85.4%) or surgical re-exploration with duraplasty (6/41, 14.6%). All CSF leakages resolved without long-term sequelae. In craniectomy patients with watertight closure (n = 224), multivariate analysis identified BMI > 30 (OR = 2.1, *P* = .02) and tumor size > 3 cm (OR = 1.9, *P* = .03) as independent risk factors.

**Table 4:** Nature of CSF and management of CSF leakages.

Type	Number of patients (n = 41)	Percentage (%)
Nature of CSF leakages		
Pseudomeningocele	30	73.2
Wound leakage	8	19.5
Rhinorrhea	3	7.3
Otorrhea	Nil	Nil
Management of CSF leakages		
Lumbar drainage	35	85.4
Surgical re-exploration with duraplasty	6	14.6

CSF = cerebrospinal fluid.

In terms of in-hospital stay, the mean in-hospital length of stay in day was 9.5 ± 1.6 in the craniotomy group while the mean in-hospital length of stay in day was 14 ± 2.1 in craniectomy group. Thus, a significant difference exists in terms of in-hospital length of stay between craniotomy and craniectomy groups (*P* < .0001). Also, in terms of hospital cost, the average cost in Chinese RMB was 58,302 ± 34.5 in the craniotomy group and 70,399 ± 55.2 in craniectomy group. Interestingly, a significant difference exists in terms of hospital cost between craniotomy and craniectomy groups (*P* < .0001). In-hospital stay and cost are shown in Table 2.

## 4. Discussion

The retrosigmoid approach for lesions at the CPA remains a critical route for neurosurgeons after it was first successfully performed by Dandy in the 1930s.^[6]^ However, this approach is still associated with high incidence of postoperative CSF leakage notwithstanding various significant alterations of the approach.^[7,11]^ In our current comparative study, we found that in retrosigmoid approach for lesions at the CPA, craniotome which involves bone replacement was crucial for the obliteration of CSF leakage in cases where the dura could not be sutured in a watertight fashion while craniectomy, which does not involve bone replacement was associated with CSF leakages. Notably, bone window size did not independently predict CSF leakage in our cohort, suggesting that dural closure quality and technique may outweigh anatomical factors.

Also, we observed significate differences in-hospital stay and cost between patient who underwent craniotome and craniectomy. Thus, craniectomy puts more burden on patients compared to craniotome. Notably, both craniotomy and craniectomy makes room for direct and continuous visualization of the dura mater while removing each bone fragment.^[9]^ Also, in both methods the thin dura layer can be steadily and meticulously stripped off the bone, thus averting dura damage which could make it challenging or impossible to achieve a watertight closure of the native dura after resecting the lesion.^[9]^ However, both methods potentially had chances of CSF leakages even when watertight closure of the native dura was totally achieved after resecting the lesion.^[9,12–15]^

We did not observe any significant difference in terms of age, sex and preoperative hydrocephalus between craniotomy and craniectomy groups. The tumor types resected included acoustic neuroma, meningioma, metastasis, glioma, hemangioblastoma, epidermoid cyst, cavernous malformation, and choroid plexus papilloma. Notably, there was no significant difference in terms of tumor type and tumor sizes between craniotomy and craniectomy groups. Comparatively, age, sex preoperative hydrocephalus, tumor type and size did not contribute to the occurrence of postoperative CSF leakage in craniotomy and craniectomy groups. Diabetic and obese patients (BMI > 30) had significantly higher CSF leak rates, likely due to impaired wound healing and elevated intra-abdominal pressure. Postoperative radiotherapy and chemotherapy, as adjunct therapies, appear to have no significant impact on CSF leak. This may be because these therapies are typically administered 1 month post-surgery, by which time most patients’ wounds have usually healed sufficiently.

Notably, a comparative study on posterior fossa tumors patients who underwent craniotomies and craniectomies with dura tears or damages after bone removal and incomplete closure of all dura revealed that CSF leak occurred in 2 % of patients who underwent craniotomy and 11.5 % patients who underwent craniectomy and a significate difference was noted between the 2 methods.^[9]^ Also, the study observed that 50% of patients with incomplete dura closure in the craniectomy group developed pseudomeningocele, even if a dura substitute was used which signifies that dura integrity is very important for successful posterior fossa surgery.^[9]^

The study above concluded that incomplete native dura closure was an independent risk factor for pseudomeningocele even though the number of patients was very small.^[9]^ Similarly, a systematic review and meta-analysis involving the rates of CSF leak or pseudomeningocele formation in posterior fossa craniotomies and craniectomies did not find a significate differences between the 2 groups despite the craniotomy group having 5.79% of CSF leakages while craniectomy group had 11.00% CSF leakages.^[7]^

Specifically, with respect to the retrosigmoid approach, previous studies reported an increased risk of leakage in patients who underwent craniectomy.^[19,20]^

Our study showed that only 3 out of 83 patients (3.6%) experienced CSF leakage in the craniotomy group, while 38 out of 262 patients (14.5%) experienced CSF leakage in the craniectomy group. We observed a significant difference in terms of CSF leakage between craniotomy and craniectomy groups (*P* = .0059). Thus, in cases where the dura cannot be sutured in watertight fashion, craniotomy may be a better option. All CSF leakages occurred during the in-hospital stay and no CSF leakages occurred 3 months after surgery or the first surgical follow-up. The nature of CSF leakages included pseudomeningocele, wound leakage, and rhinorrhea. No otorrhea was observed. Notably, all CSF leakages were managed with lumbar drainage or surgical re-exploration with duraplasty. All CSF leakages resolved without long-term sequelae

Mechanically, the replaced bone flap serves as a rigid support over the dura closure and prevents the dura sutures from been torn out by increase pressure from postoperative CSF accumulation within the posterior fossa.^[9,12]^ Thus, any small defect along the dura closure will not enlarge, and CSF is less likely to leak out and accumulate in the subcutaneous plains, or flow out of the skin.^[9,12]^ Also, the replaced bone flap restores the natural anatomic support for timely reattachment of the neck muscle layer. Thus, it becomes total or nearly possible for CSF to accumulate in a deep dead space between the dura and the muscles.^[9,12]^

Notably, CSF leakage have been implicated in complications like meningitis, brain abscess as well as hydrocephalus.^[16]^ We did not observe any significate difference in intracranial infection between the craniotomy and craniectomy groups. Besides poor dura closure, factors such as secondary cerebellar swelling due intraoperative retraction with dehiscence of the dura stitches and poor general condition of the patient also contributes to CSF leakage after posterior fossa or retrosigmoid surgical approach.

The most common triggers of CSF leakage from the neurosurgical point of view are inadequate or inappropriate sealing of the unroofed posterior wall of internal acoustic meatus with opened petrous air cells as well as direct drainage to the middle ear and opened mastoid air cells with communication to the middle ear.^[10]^

Preventatively, bone wax has been used to occlude the opened mastoid cells but was associated with failure, allergic reactions in the mastoid and development of foreign body granuloma in the CPA.^[21–23]^ Also, fibrin glue or routine postoperative prophylactic continuous external lumbar CSF drain were helpful in reducing incidence of CSF leakages.^[13–15]^ Nevertheless, some authors did not find benefit in the usage of prophylactic continuous external lumbar CSF drain to prevent or reduce incidence of postoperative CSF leakages.^[24]^ Moreover, it delayed early mobilization of the patient with risk of more postoperative hitches.^[10]^

Interestingly, biomaterial like polymethylmethacrylate cement, hydroxyapatite bone cement as well as demineralized bone matrix were effective in reducing the CSF leakages after a retrosigmoid craniectomy.^[25–28]^ Nevertheless, performing a reopening procedure significantly increased the risk of CSF leakage in the postoperative course of patients with initial CSF leakages.^[29,30]^ Notably, 50% of patients who underwent reoperation because of CSF leakage after retrosigmoid craniectomy developed significantly higher CSF leakage.^[31]^ Interestingly, all the patients who developed CSF leakages were managed conservatively until their leakages stopped. Notably, no reoperations or external ventricular drainages were not performed in any of the cases that developed CSF leakages.

In teams of hospital stay, an earlier study reported a mean in-hospital length of stay of 9.3 days for craniotome group and 11.8 days for craniectomy group.^[9]^ They observed no significant difference between the 2 groups.^[9]^ We observed a mean in-hospital length of 9.5 ± 1.6 days in our craniotomy group and 14 ± 2.1 days in our craniectomy group. Interestingly, a significant difference exists between our craniotomy and craniectomy groups (*P* < .0001). Also, in terms of hospital cost, the average cost in craniotomy group was less than craniectomy group. Interestingly, a significant difference exists in terms of hospital cost between craniotomy and craniectomy groups (*P* < .0001). This study has limitations because it is a single-center retrospective type and does not have the rigor of the prospective study. A multicenter prospective study will be more conclusive.

## 5. Conclusions

Comparatively, craniotome was crucial in the obliteration of CSF leakage in cases where the dura could not be sutured in a watertight fashion while craniectomy was associated with CSF leakages. Also, the were a significate differences in-hospital stay and cost between patient who underwent craniotome and craniectomy. Thus, craniectomy puts more burden on patients compared to craniotome.

## Author contributions

**Conceptualization:** Min Tang, Seidu A. Richard, Zhigang Lan.

**Data curation:** Min Tang, Zhigang Lan.

**Formal analysis:** Min Tang, Seidu A. Richard, Zhigang Lan.

**Funding acquisition:** Zhigang Lan.

**Investigation:** Min Tang, Seidu A. Richard, Zhigang Lan.

**Methodology:** Min Tang, Seidu A. Richard, Zhigang Lan.

**Resources:** Min Tang.

**Software:** Seidu A. Richard, Zhigang Lan.

**Supervision:** Zhigang Lan.

**Writing – original draft:** Seidu A. Richard.

**Writing – review & editing:** Min Tang, Seidu A. Richard, Zhigang Lan.
